# Analysis of Risk Factors for Augmented Vertebral Refracture After Percutaneous Kyphoplasty in Osteoporotic Vertebral Compression Fractures

**DOI:** 10.3390/jcm14020329

**Published:** 2025-01-08

**Authors:** Yonghao Wu, Shuaiqi Zhu, Yuqiao Li, Chenfei Zhang, Weiwei Xia, Zhenqi Zhu, Kaifeng Wang

**Affiliations:** Department of Spinal Surgery, Peking University People’s Hospital, No.11 Xizhimen South Street, Beijing 100044, China; wuyonghao1998@163.com (Y.W.); 2311210331@stu.pku.edu.cn (S.Z.); drliyq@163.com (Y.L.); zhangchenfei01@163.com (C.Z.); wx@bjmu.edu.cn (W.X.); zhuzhenqi2000@vip.sina.com (Z.Z.)

**Keywords:** osteoporosis, postoperative complications, vertebral body, vertebroplasty

## Abstract

**Objectives**: The aim of this study was to investigate the incidence of vertebral refractures following percutaneous kyphoplasty (PKP) and to explore risk factors for augmented vertebral refractures, thereby assisting spinal surgeons in clinical practice. **Methods**: We analyzed the records of 495 patients with single-segment osteoporotic vertebral compression fractures (OVCFs) who were treated with single-entry PKP at our institution from March 2016 to August 2022. Univariate analysis, binary logistic regression, and ROC curve analysis were performed to determine potential risk factors, independent risk factors, and discrimination ability. **Results**: A total of 168 patients were included in the study, with a median follow-up duration of 7.00 months. In total, 143 patients did not experience vertebral refracture after surgery, while 25 patients did, including 22 augmented vertebral fractures and 3 adjacent vertebral compression refractures. The correction rate of the Cobb angle (*p* < 0.001; OR = 1.070) and postoperative anti-osteoporosis treatment (*p* = 0.002; OR = 0.021) were independently associated with augmented vertebral refracture. The ROC curves showed that these variables demonstrated satisfactory predictive values for augmented vertebral refracture. **Conclusions**: A high degree of restoration of the Cobb angle was the factor contributing to vertebral refracture after PKP. Conversely, postoperative anti-osteoporosis treatment was observed to be a protective factor against subsequent vertebral refracture.

## 1. Introduction

Osteoporosis (OP) is a systemic disease characterized by bone loss and increased fracture risk, and osteoporotic vertebral compression fractures (OVCFs) are the most common type of fracture caused by osteoporosis. OP and OVCFs have become increasingly common worldwide. In the United States during the years 2013 to 2014, OP was reported to impact between 6% and 11% of adults aged 50 years and older [1]. Globally, OVCFs affect nearly 50% of women and around 20% of men over the age of 50 [2]. In Europe, the incidence of vertebral fractures among women is 10.7/1000 per year, while the incidence among men is 5.7/1000 per year [3]. In the United Kingdom, approximately 86,000 new cases of osteoporotic vertebral fractures are reported annually [4]. In Thailand, approximately one-third of postmenopausal women are affected by undiagnosed vertebral fractures [5]. The prevalence of vertebral fractures in postmenopausal Caucasian women ranges from 15% to 35%. Additionally, the prevalence of vertebral fractures among Spanish men is reported to be 21.3%. In China, the prevalence of OP among individuals aged 50 years and above was 32% in 2018 [6], and the incidence rate of OVCFs was 152.13 per 100,000 person-years in urban China in 2017 [7].

**Figure 1:**
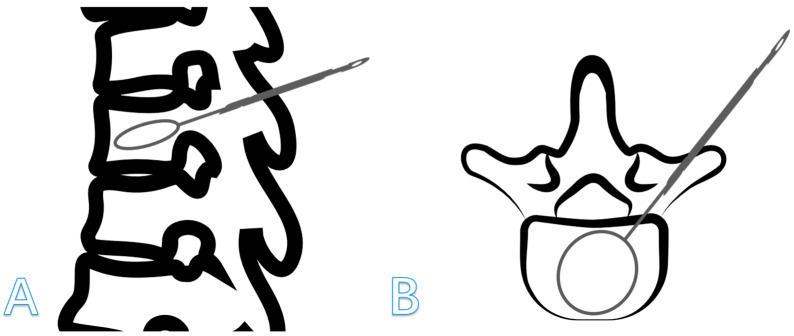
Schematic images of balloon dilatation in percutaneous kyphoplasty. (**A**) Sagittal plane; (**B**) Transverse Plane.

Vertebroplasty, including percutaneous vertebroplasty (PVP) and percutaneous kyphoplasty (PKP) (Figure 1), is a well-known and efficacious treatment for alleviating patients’ pain, correcting kyphosis, and restoring vertebral body height [8,9]. However, vertebral refractures remain a common complication following vertebroplasty [10]. In particular, compared with PVP, PKP has superior efficacy in correcting the Cobb angle and restoring the vertebral height, which is usually considered an advantage of PKP [11]. However, numerous scholars contend that an effective correction rate of the Cobb angle and effective recovery of vertebral height are associated with an increased risk of vertebral refracture [12]. For example, Sahinturk [13], through a 10-year follow-up study, indicated that the incidence of augmented vertebral fractures after PKP is higher than after PVP, with the rate of augmented vertebral fractures post-PKP being 27.5%, compared to 13.3% post-PVP. Various signs indicated that the advantages of PKP over PVP may have shifted towards a potential risk of vertebral refracture. Consequently, spinal surgeons must consider postoperative vertebral refractures when performing PKP treatment.

Our study aims to investigate the incidence of vertebral refractures following PKP and to explore risk factors for augmented vertebral refractures as an example. This research will provide valuable references and guidance for spinal surgeons in preventing vertebral refractures, thereby bolstering their confidence in conducting PKP treatments and facilitating the broader application of PKP in clinical practice.

The schematic images presented herein are composed of vector graphics, sourced from the material library [https://www.iconfont.cn/ (accessed on 25 December 2024)].

## 2. Materials and Methods

### 2.1. General Data

**Figure 2:**
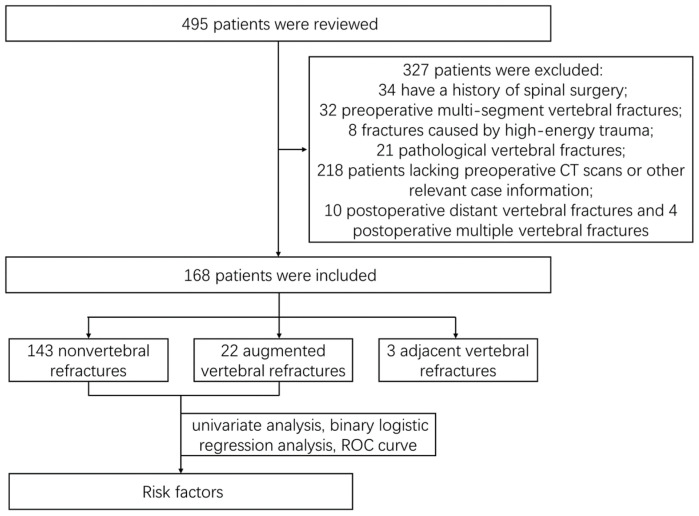
Flowchart of the study.

The records during hospitalization and outpatient follow-up of 495 patients with single-segment OVCFs who underwent single-entry PKP at the Department of Spinal Surgery, Peking University People’s Hospital, between March 2016 and August 2022, were extracted, and 182 patients were eventually included (Figure 2). The Medical Ethics Committee of Peking University People’s Hospital approved the study protocol, and the approval number was 2024PHB007-001. The study was conducted in accordance with the ethical standards of the Helsinki Declaration, and all participants signed an informed consent form.

The inclusion criteria were as follows: (1) the presence of a fragility fracture resulting from minor trauma, such as falling from a height equal to or lower than that of standing position; (2) a fresh single-level vertebral fracture, accompanied by back pain; (3) patients who had preoperative computed tomography (CT) and could be diagnosed with OP with Hounsfield Units (Hus) on CT at upper lumbar levels ≤ 110 [14,15]; and (4) magnetic resonance imaging (MRI) indicating a new vertebral fracture, with the vertebra showing a low signal on T1WI, high signal on T2WI, and fat suppression sequence.

The exclusion criteria were as follows: (1) a history of spinal surgery, including open spinal surgery, minimally invasive surgery, or vertebroplasty; (2) vertebral fractures involving multiple segments; (4) fractures caused by high-energy trauma; (5) pathological vertebral fractures; (6) patients lacking preoperative CT scans or other relevant case information; and (7) postoperative distant vertebral fractures or multiple vertebral fractures.

Vertebral refracture was diagnosed based on a reduction of 4 mm or more in the height of the vertebral body [16,17,18] within the first three days post-surgery.

In our study, postoperative single vertebral refractures were classified into three types: augmented vertebral refractures and adjacent vertebral compression refractures (AVCFs). Furthermore, AVCFs can be further categorized into upper adjacent vertebral compression refractures and lower adjacent vertebral compression refractures.

### 2.2. Treatment Method

Percutaneous kyphoplasty was carried out by two highly experienced senior surgeons, each with over a decade of expertise in PKP at our institution. The patient was positioned prone on the surgical table and underwent thorough disinfection prior to surgery. X-ray imaging was subsequently employed to accurately identify the body surface corresponding to the pedicle of the fractured vertebra. Following the successful administration of local anesthesia using 2% lidocaine, a 0.5 cm incision was made on the body surface. A puncture needle with a cannula was inserted into the surface of the pedicle of the fractured vertebra. Before the cannulated puncture needle was inserted into the pedicle, a C-arm machine was used to confirm the precise positioning of the puncture needle, and the surgeons made some necessary adjustments. The surgeons inserted the cannulated puncture needle into the pedicle and removed the needle core. The cannula was subsequently maintained, and the puncture needle was removed. The guide needle was subsequently inserted into approximately two-thirds of the forepart of the fractured vertebral body. While maintaining the guide needle in a stationary position, the cannula was extracted and replaced with a larger cannula to widen the pedicle channel. The guide needle was then removed, and the cannula remained. The balloon dilator was inserted into the established pedicle channel. Contrast media was infused into the balloon via a manometer-equipped syringe, and its location and expansion were monitored using the X-ray of a C-arm machine while keeping an eye on the pressure levels. Upon reaching 16 kPa and encountering considerable resistance during inflation, decompression was performed, and then, the balloon was withdrawn. A specialized catheter was employed to deliver semisolid bone cement into the vertebral body via the pedicle channel. The C-arm machine was subsequently used to verify the position of the cement after solidification. Finally, the wound was dressed.

### 2.3. Evaluation Index

The evaluation indices used were as follows: age, gender, body mass index (BMI), underlying disease, history of fracture at other sites, Hus on CT at upper lumbar levels, volume of cement, postoperative anti-osteoporosis treatment, primary fractured vertebral segment, intravertebral vacuum cleft (IVC), preoperative and postoperative distance between the bone cement and anterior edge of the vertebral body, sagittal position of cement filling, contact between the bone cement and endplate, distance between the bone cement and vertebral endplates, collapse of the vertebral body, restoration rate of the vertebral anterior margin height, correction rate of the Cobb angle, bone–cement distribution score, and bone cement leakage.

**Figure 3:**
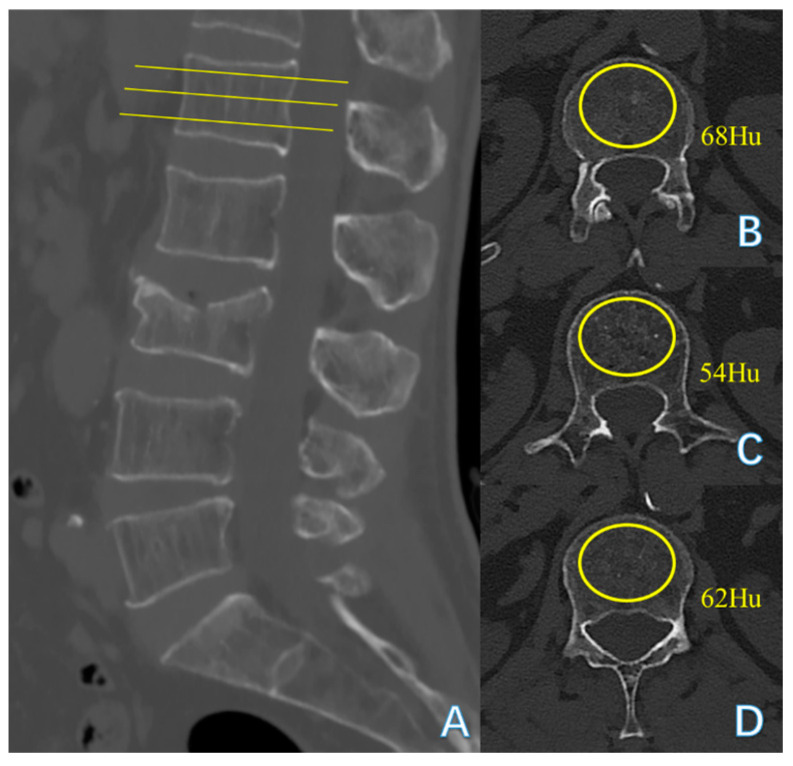
A 70-year-old male patient with compression fracture of L3: (**A**) midsagittal plane; (**B**) inferior to the upper endplate; (**C**) in the middle of the vertebral body; (**D**) superior to the lower endplate. The Hu on CT was (68 + 54 + 62)/3 = 61.33.

**Figure 4:**
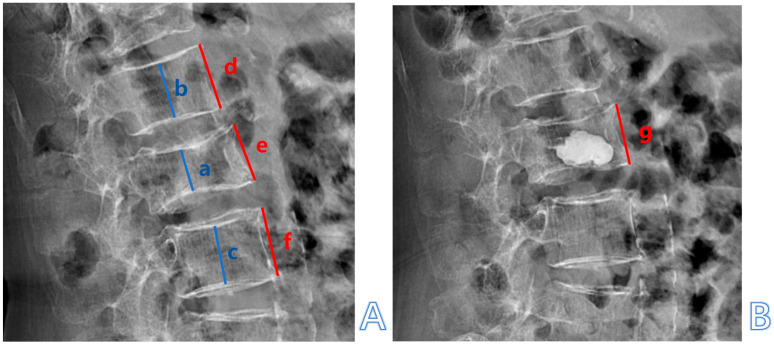
An 89-year-old female patient with an OVCF at the L2 level. (**A**) Preoperative lateral X-ray imaging. (**B**) Lateral X-ray imaging taken on the postoperative day. a. Preoperative fractured vertebral body height; b. upper adjacent vertebral body height; c. lower adjacent vertebral body height; collapse of the vertebra = 1 − 2 × a/(b + c). e. Postoperative fractured vertebral anterior margin height; d. upper adjacent vertebral anterior margin height; f. lower adjacent vertebral anterior margin height; g. preoperative fractured vertebral anterior margin height; recovery rate of vertebral anterior margin height = 2 × (e − g)/(d + f).

**Figure 5:**
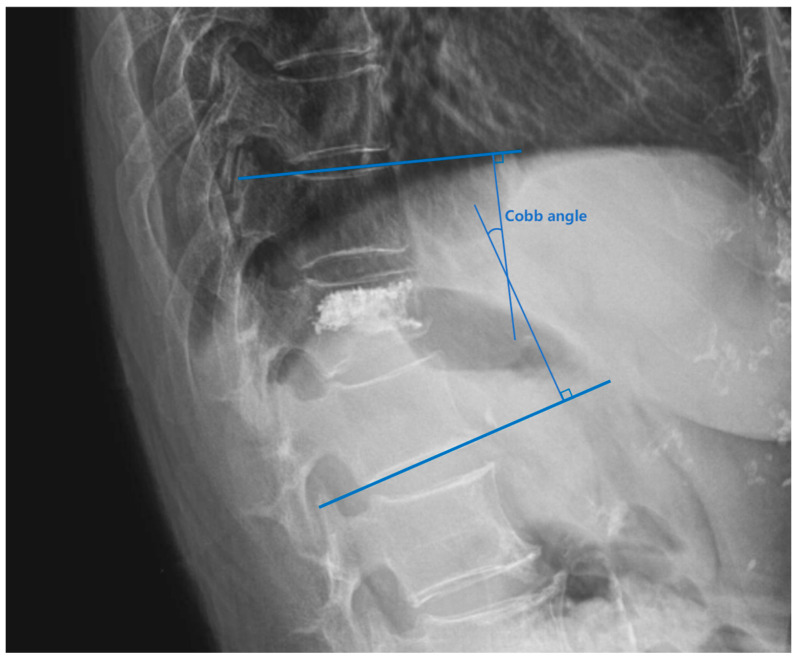
Method for measuring the Cobb angle. The Cobb angle in this study was defined as the angle between the superior endplate of the superior vertebra of the fractured vertebra and the inferior endplate of the inferior vertebra of the fractured vertebra.

**Figure 6:**
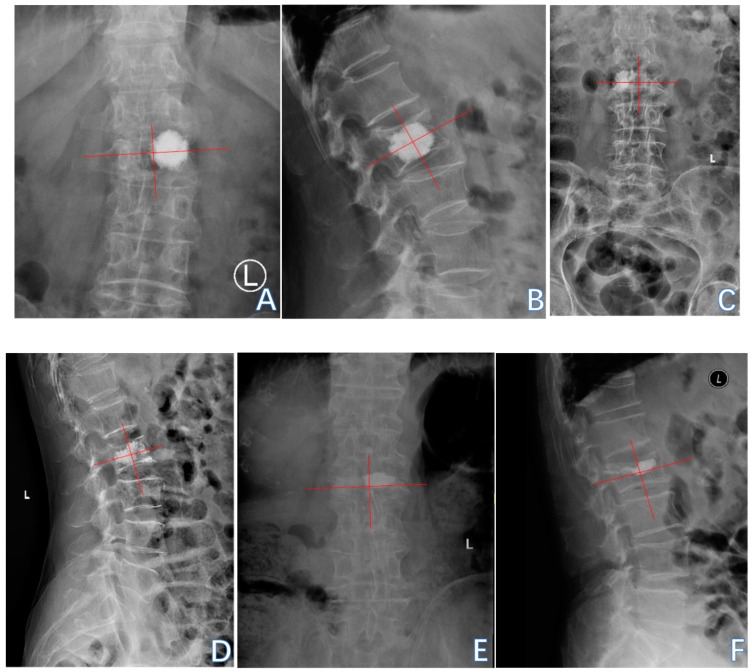
(**A**,**B**): An 80-year-old female patient diagnosed with an OVCF at L2 underwent PKP. The bone–cement distribution score was calculated as 2 + 4 + 2 = 8. (**C**,**D**): A 67-year-old female patient diagnosed with an OVCF at L2 underwent PKP. The bone–cement distribution score was calculated as 2 + 4 + 2 = 8. (**E**,**F**): An 80-year-old male patient diagnosed with an OVCF at L2 underwent PKP. The bone–cement distribution score was calculated as 1 + 2 + 1 = 4.

The thoracolumbar junction comprised T11-L2. The Hu on patients’ preoperative CT of L1 vertebral body was measured by the region of interest (ROI) to diagnose OP and by the L2 vertebral body when L1 was fractured. The ROI, which was confined to the cancellous bone area to reduce the influence from the cortical bone and placed far away from certain structures such as the venous plexus was expanded to as large as possible in the three separate locations of axial images (inferior to the upper endplate, in the middle of the vertebral body, and superior to the lower endplate) (Figure 3) [19].The sagittal position of cement filling [20] could be determined through a lateral X-ray of the spine, taken on the first day after PKP, which was divided into 1/3 of the vertebral body filling, 2/3 of the vertebral body filling, and the whole vertebral body filling. The restoration rate of the vertebral anterior margin height [21] was calculated as 2 × (postoperative fractured vertebral anterior margin height—preoperative fractured vertebral anterior margin height)/(upper adjacent vertebral anterior margin height + lower adjacent vertebral anterior margin height) (Figure 4). The postoperative fractured vertebral anterior margin height was measured using lateral X-ray imaging of the spine on the 1st day after PKP. The degree of collapse of the vertebra [22] was defined as [1 − 2 × preoperative fractured vertebral body height/(upper adjacent vertebral body height + lower adjacent vertebral body height)] (Figure 4). The heights of the vertebrae were measured at the location of maximum collapse. The correction rate of the Cobb angle [23] was calculated as (preoperative Cobb angle—postoperative Cobb angle)/preoperative Cobb angle (Figure 5). The bone–cement distribution score was calculated using Liu’s method [24]; that is, the surgical vertebra was divided into four quadrants on anteroposterior and lateral X-ray films. If one-third or more of a quadrant was filled with bone cement, it was considered one point, and if there was contact between the bone cement and either the upper or lower endplate, each contact was considered one point. A total score of 10 points was obtained (Figure 6).

### 2.4. Statistical Analysis

The samples were divided into a vertebral refracture group and a nonvertebral refracture group. We analyzed the proportion of fracture types among patients who experienced vertebral refractures, with a particular emphasis on identifying the risk factors associated with augmented vertebral refractures. The data analysis was conducted utilizing SPSS version 26 (IBM, Armonk, NY, USA). The possible risk factors associated with vertebral refracture after PKP were obtained through univariate analysis. The Shapiro–Wilk test was used to detect the normality of continuous variables. The independent sample *t* test was used for analyzing continuous variables with normal distributions, the Mann–Whitney u test for continuous variables with non-normal distributions, and the chi-square test for categorical variables. Binary logistic regression was used to analyze the possible risk factors and obtain the independent risk factors for vertebral refracture. Finally, the discriminant ability was evaluated by the area under the curve (AUC) of the receiver operating characteristic (ROC) curve. A *p* value < 0.05 was considered to indicate statistical significance.

## 3. Results

### 3.1. General Situation

After 6–42 months of follow-up, 168 patients were involved, with a median of 7.00 months of follow-up, a mean age of 73.64 ± 8.446 years, and 77.38% females. In more detail, 143 patients did not experience vertebral refracture after operation, whereas 25 patients experienced vertebral refracture after surgery, including 22 augmented vertebral refractures and 3 AVCFs. The duration between surgery and the occurrence of vertebral refracture ranged from 0.5 to 38 months, with 6.73 months on average. In the following sections, we focus on exploring the risk factors associated with augmented vertebral refracture.

### 3.2. Augmented Vertebral Refractures

The study subjects were divided into two groups: the nonvertebral refracture group (n = 143) and the augmented vertebral refractor group (n = 22). Using Augmented vertebral refractures as a case study, we explored possible and independent risk factors through univariate analysis and binary logistic regression analysis, validating the predictive efficacy of each factor using the ROC curve.

#### 3.2.1. Univariate Analysis

Through univariate analysis, we identified possible risk factors related to augmented vertebral refracture after PKP, including a low Hu at the upper lumbar levels, thoracolumbar junction fracture, preoperative IVC, a high restoration rate of the vertebral anterior margin height, a low postoperative Cobb angle, a high correction rate of the Cobb angle, non-cement–endplate contact, a long distance between the bone cement and vertebral endplates, a low bone–cement distribution score, bone cement leakage, and an absence of postoperative anti-osteoporosis treatment (Table 1).

#### 3.2.2. Binary Logistic Regression Analysis

Binary logistic regression revealed that restoration of the Cobb angle (OR = 1.070; 95% CI 1.034~1.109; *p* < 0.001) and postoperative anti-osteoporosis treatment (OR = 0.021; 95% CI 0.002~0.253; *p* = 0.002) were independently associated with augmented vertebral refracture after PKP (Table 2). Specifically, postoperative anti-osteoporosis treatment emerged as a protective factor against such occurrences, with an OR < 1, as did restoration of the Cobb angle, with OR > 1.

#### 3.2.3. Receiver Operating Characteristic (ROC) Curve

**Figure 7:**
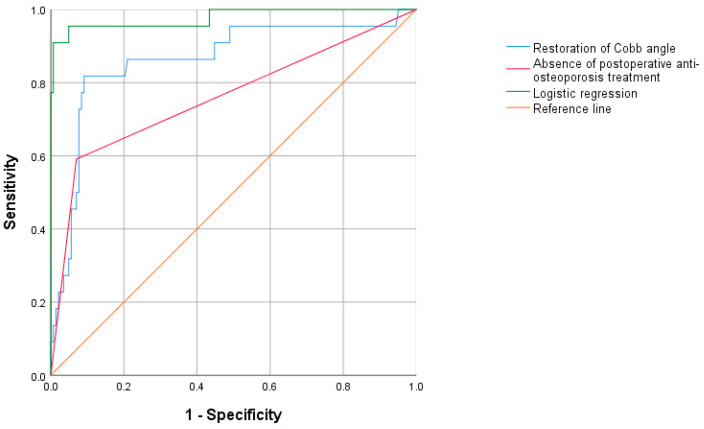
Receiver operating characteristic curves for the predictive performance of independent risk factors associated with augmented vertebral refracture following PKP.

ROC curve analysis was employed to evaluate the predictive performance of the restoration of the Cobb angle and an absence of postoperative anti-osteoporosis treatment (Figure 7). The optimal cutoffs, along with the corresponding sensitivity, specificity, and AUC values, are presented in Table 3.

Restoration of the Cobb angle and an absence of postoperative anti-osteoporosis treatment were of satisfactory predictive value for augmented vertebral refracture, with AUCs of 0.863 and 0.760, respectively. In particular, the AUC of the binary logistic regression model was 0.977, indicating that it had a strong predictive ability for augmented vertebral refractures.

## 4. Discussion

Vertebral refracture was a frequently encountered complication following vertebroplasty. Previous studies have revealed inconsistent incidences of vertebral refracture, ranging from 0.56% to 33.5% [12,25]. In this retrospective study involving 168 single-segment OVCFs, the overall incidence of vertebral refracture following single-entry PKP was 14.88%, ranging from 0.5 to 38 months, with 6.73 months on average. Specifically, the incidence of augmented vertebral refracture was 13.33%.

A high correction rate of the Cobb angle was identified as a risk factor for bone cement leakage [26], bone cement displacement [21], and vertebral refracture [12]. In this study, the recovery rates of the vertebral anterior margin height and the correction rate of the Cobb angle in the vertebral body refracture group were significantly greater than those in the nonvertebral refracture group (*p* < 0.05) (Table 1). Han [27] and Dai [12] reported that a high restoration rate of the vertebral anterior margin height might exert greater pressure on adjacent vertebrae, which is a risk factor for AVCF following vertebroplasty. Zhu [28] suggested that a high level of restoration of the vertebral anterior margin height and Cobb angle correction may increase tension in paraspinal tissue and cause osteonecrosis in the augmented vertebral body, ultimately leading to refracture.

In addition, Cao [29] examined the postoperative Cobb angle and sagittal spino-pelvic parameters of 90 patients with OVCFs who underwent PKP and suggested that the correction rate of the Cobb angle after PKP was closely associated with not only the spinal segmental balance but also the overall sagittal balance of the spine. Excessive recovery of the Cobb angle might result in rigid intervertebral connections and sagittal spinal imbalance [20,30], potentially leading to distant vertebral refracture post-PKP. It is essential for spinal surgeons to bear in mind that PKP is a type of orthopedic technique, but surgeons should not aim for anatomical correction of the kyphosis nor an excessive correction rate of the Cobb angle. Therefore, attention should be given not only to restoring spinal segmental balance but also to improving the overall sagittal balance of the spine in clinical practice.

Generally speaking, the T-scores measured by dual-energy X-ray absorptiometry (DXA) have been considered the gold standard of bone mineral density (BMD) assessment [31], and a DXA T-score ≤ −2.5 was the gold standard for diagnosing OP [32]. Regrettably, DXA has little application in orthopedic clinical practice, which also has not been included as a standard examination for hospitalized patients at our institution. The Hu on CT at the upper lumbar levels ≤ 110 was also widely recognized as a diagnostic tool for OP [14]. Therefore, the Hu on CT at the upper lumbar levels, considered a “surrogate marker” [19] for BMD, was used to evaluate BMD in this study. Consistent with previous studies, a low Hu was a possible risk factor for augmented vertebral refractures after PKP in OVCFs (Table 2).

A low BMD was closely related to the occurrence of vertebral refractures following PKP in OVCFs. Postoperative anti-osteoporosis therapy has been shown to effectively mitigate bone loss in patients, slow the progression of osteoporosis, and prevent vertebral body fractures after PKP. The OR of postoperative anti-osteoporosis therapy was <1 in this study (Table 2), indicating that it served as a protective factor against vertebral body refracture. Zhang [33] reported that the absence of postoperative anti-osteoporosis treatment constituted the primary risk factor for vertebral refracture after PKP. In their investigation, 51% of patients with nonvertebral refracture received anti-osteoporosis treatment, whereas only 22% of patients with vertebral refracture did. Moreover, standardized postoperative anti-osteoporosis treatment plays a crucial role in improving patient prognosis and survival rates [34].

Compared to PVP, during the process of PKP, balloon dilation compacts the surrounding cancellous bone and creates a “low-pressure cavity “ at the site of expansion. Under the influence of the obstructive effect from tightly packed cancellous bone and the suction effect of this low-pressure cavity, post-PKP cement exhibits a tendency towards a lump-like distribution, making it difficult to adequately diffuse within the vertebral body [35]. While this may help prevent postoperative bone cement leakage, it simultaneously results in low bone–cement distribution scores and inadequate contact between the cement and endplates, which are possible risk factors for augmented vertebral fractures following PKP (Table 1). Furthermore, compared to PVP, PKP is associated with higher rates of vertebral height restoration and Cobb angle recovery [11], contributing to vertebral refractures.

Some novel vertebral augmentation systems have been improved based on the operational characteristics of PKP procedures, reducing the incidence of postoperative vertebral refractures. The first is KIVA vertebral augmentation. During the surgical process under the KIVA system [36], the operator uses an external handle to push a nitinol coil guide wire through a deployment cannula into cancellous bone. Subsequently, a polyether ether ketone (PEEK) implant, incorporating 15% barium sulfate to enhance its radiopacity, is positioned over the coil to create nested cylindrical columns, continuing this process until the targeted restoration of the fractured vertebral height is accomplished. Upon the retraction of the coil, poly (methyl methacrylate) (PMMA) cement is subsequently administered through the inner channel of the PEEK implant. The PEEK implant directly penetrates osteoporotic cancellous bone to create space for PMMA cement implantation, rather than compressing and displacing cancellous bone with an inflatable balloon as in PKP [37]. This approach eliminates resistance from surrounding pushed dense cancellous bone and reduces the suction effect from the low-pressure cavity, theoretically allowing for a more uniform diffusion of bone cement and practically lowering the incidence of vertebral refractures [38]. The second is percutaneous curved vertebroplasty (PCVP). PCVP is an improved unipedicular approach to vertebroplasty, which utilizes a flexible injection cannula to effectively deliver bone cement into the contralateral vertebra through a unilateral access route, thereby achieving a uniform distribution of the cement [39]. Zhou [40], conducting a prospective controlled study involving 94 patients, reported that the incidence of vertebral fractures in OVCFs after PCVP was significantly lower than that observed in patients following bilateral PKP. The third method is cement-augmented pedicle screw fixation (CAPSF). CAPSF combines bone cement augmentation with traditional pedicle screw fixation, effectively restoring vertebral height, enhancing vertebral stiffness, and improving the internal stability of the vertebrae [41]. This technique significantly reduces the incidence of screw loosening and displacement following traditional pedicle screw fixation [42], as well as the occurrence of refractures in cases that are solely treated with bone cement augmentation. Last, but not least, the conservative treatment with brace should not be overlooked. For patients with mild vertebral re-compression and minimal pain, treatment through bracing rather than surgical intervention can be effective. In this study, all 39 patients with vertebral refractures underwent conservative treatment without further surgery. Wearing a brace has been shown to alleviate residual pain following vertebroplasty, enhance the quality of life for patients, and concurrently reduce the incidence of postoperative vertebral refractures [25].

This study also had the following limitations: Firstly, compared with the extensive application of MRI and X-ray, numerous patients with OVCFs lacked preoperative CT because of its fewer advantages in the diagnosis and condition evaluation of OVCF, which was the main reason for the high rate of lost visits (313/495, 63.2%) in this study. Secondly, the follow-up period is relatively short. In this study, patients were followed for 6 to 42 months, with a median follow-up time of only 7 months. This may have excluded some patients who could experience vertebral refractures in the future from the refracture group, leading to an underestimated incidence of refractures. Thirdly, this study defines a vertebral refracture as a decrease in vertebral height of at least 4 mm, which may result in the oversight of subtle endplate or hairline fractures. Finally, this study was a retrospective study based on outpatient follow-up, so it was difficult to follow up regularly and improve the subjective pain scores, such as the preoperative and postoperative visual analogue scale (VAS) and Oswestry disability index (ODI). Prospective, multi-center, and large-sample studies are needed in the future to further clarify the risk factors of vertebral refracture after PKP. In future research, we plan to include preoperative and postoperative MRI, X-rays, and CT scans of prospectively enrolled patients in our assessment. Additionally, regular follow-ups will be conducted to evaluate their VAS and ODI scores, which will also contribute to reducing the lost to follow-up rate.

## 5. Conclusions

A low Hu at the upper lumbar levels, thoracolumbar junction fracture, preoperative IVC, a high restoration rate of the vertebral anterior margin height, a low postoperative Cobb angle, a high correction rate of the Cobb angle, non-cement–endplate contact, a high distance between bone cement and vertebral endplates, a low bone–cement distribution score, bone cement leakage, and an absence of postoperative anti-osteoporosis treatment are possible risk factors for augmented vertebral refracture after PKP. Among these factors, a high correction rate of the Cobb angle is the main factor contributing to vertebral refracture after PKP. Conversely, postoperative anti-osteoporosis treatment is observed to be a protective factor against subsequent vertebral refracture.

## Figures and Tables

**Table 1:** Univariate analysis of augmented vertebral refracture after PKP in OVCFs.

	Nonvertebral Refracture Group (n = 143)	Augmented Vertebral Refracture Group (n = 22)	χ^2^/t Value/z Value	*p* Value
Age (years)	73.000 (65.500, 80.000)	78.500 (67.500, 81.75)	−1.831	0.067 *
Gender			0.343	0.558
Male [n (%)]	31 (21.7%)	6 (27.3%)		
Female [n (%)]	112 (78.3%)	16 (72.7%)		
BMI (kg/cm^2^)	24.358 ± 3.469	24.024 ± 3.709	0.400	0.489
Underlying diseases				
Hypertension [n (%)]	65 (45.5%)	10 (45.5%)	<0.001	1.000
Diabetes [n (%)]	30 (21.0%)	5 (22.7%)	0.035	0.852
Cardiovascular and cerebrovascular diseases [n (%)]	32 (22.4%)	8 (36.4%)	2.031	0.154
History of fracture at other sites [n (%)]	22 (15.4%)	3 (13.6%)	<0.001	1.000
Hu at upper lumbar levels	83.000 (68.000, 100.000)	66.000 (61.250, 80.250)	−3.110	0.002 *
Primary fractured vertebral segment			7.259	0.027
Thoracic segment [n (%)]	14 (9.8%)	1 (4.5%)		
Thoracolumbar junction (T11-L2) [n (%)]	109 (76.2%)	21 (95.5%)		
Lumbar segment [n (%)]	20 (14.0%)	0		
Preoperative IVC	91 (63.6%)	22 (100.0%)	10.057	0.002
Preoperative vertebral height (cm)	1.559 ± 0.410	1.514 ± 0.380	0.489	0.502
Collapse of vertebral body (%)	24.301 (14.643, 34.963)	34.379 (16.520, 44.591)	−1.544	0.123 *
Preoperative vertebral anterior margin height (cm)	1.811 ± 0.478	1.748 ± 0.470	0.573	0.518
Postoperative vertebral anterior margin height (cm)	1.996 ± 0.465	2.233 ± 0.461	−2.229	0.578
Recovery rate of vertebral anterior margin height (%)	5.904 (1.099, 15.621)	17.068 (9.541, 33.189)	−3.554	<0.001 *
Preoperative Cobb angle (°)	14.000 (8.800, 19.000)	14.650 (10.550, 21.775)	−0.988	0.323 *
Postoperative Cobb angle (°)	12.700 (7.900, 18.400)	7.500 (4.325, 13.150)	−2.495	0.013 *
Restoration of Cobb angle (%)	4.368 (1.917, 12.050)	43.316 (36.996, 56.754)	−5.474	<0.001 *
Volume of cement (mL)	2.500 (2.250, 3.000)	2.500 (2.500, 2.500)	−1.077	0.281 *
Sagittal position of cement filling			4.852	0.088
1/3 of vertebral body [n (%)]	11 (7.7%)	2 (9.1%)		
2/3 of vertebral body [n (%)]	45 (31.5%)	12 (54.5%)		
Whole vertebral body [n (%)]	87 (60.8%)	8 (36.4%)		
Contact between bone cement and endplate			18.474	<0.001
Non-cement–endplate contact [n (%)]	8 (5.6%)	3 (13.6%)		
Contact with upper vertebral endplates [n (%)]	25 (17.5%)	11 (50.0%)		
Contact with lower vertebral endplates [n (%)]	40 (28.0%)	6 (27.3%)		
Contact with upper and lower vertebral endplates [n (%)]	70 (49.0%)	2 (9.1%)		
Distance between bone cement and vertebral endplates (cm)	0.160 (0, 0.430)	0.380 (0.203, 0.598)	−3.123	0.002 *
Bone–cement distribution score	7.000 (5.000, 8.000)	5.000 (4.250, 6.000)	−3.761	<0.001 *
Bone cement leakage			12.696	0.013
No leakage [n (%)]	99 (69.2%)	11 (50.0%)		
Anterior leakage [n (%)]	17 (11.9%)	4 (18.2%)		
Posterior leakage [n (%)]	6 (4.2%)	4 (18.2%)		
Lateral leakage [n (%)]	6 (4.2%)	3 (13.6%)		
Disc leakage [n (%)]	15 (10.5%)	0		
Postoperative anti-osteoporosis treatment	133 (93.0%)	9 (40.9%)	43.139	<0.001

* *p* value was obtained by Mann–Whitney U test.

**Table 2:** Binary logistic regression analysis of augmented vertebral refracture after PKP in OVCFs.

	B Value	Standard Error	Wald Value	*p* Value	OR	95% CI
Hu at upper lumbar levels	−0.036	0.024	2.269	0.132	0.965	0.920~1.011
Thoracolumbar junction	16.777	>2.000	<0.001	0.995	>1.000	-
Preoperative IVC	21.605	>2.000	<0.001	0.996	>1.000	-
Recovery rate of vertebral anterior margin height	0.026	0.100	0.070	0.791	1.027	0.844~1.249
Postoperative Cobb angle	−0.187	0.218	0.741	0.389	0.829	0.541~1.271
Restoration of Cobb angle	0.068	0.018	14.467	<0.001	1.070	1.034~1.109
Contact between bone cement and endplate						
Non-cement–endplate contact *			1.057	0.787		
Contact with upper vertebral endplates	2.541	12.172	0.044	0.835	12.696	-
Contact with lower vertebral endplates	5.361	12.478	0.185	0.667	212.964	-
Contact with upper and lower vertebral endplates	6.088	12.771	0.227	0.634	440.691	-
Distance between bone cement and vertebral endplates	1.166	1.154	1.020	0.312	3.209	0.334~30.841
Bone–cement distribution score	−0.311	0.697	0.199	0.655	0.733	0.187~2.870
Bone cement leakage						
No leakage *			2.319	0.677		
Anterior leakage	5.190	4.306	1.453	0.228	179.557	0.039~830,739.907
Posterior leakage	1.435	3.784	0.144	0.704	4.200	0.003~6986.566
Lateral leakage	5.791	3.824	2.293	0.130	>1.000	0.182~588,576.042
Disc leakage	−14.362	>2.000	<0.001	0.999	<1.000	-
Postoperative anti-osteoporosis treatment	−3.878	1.277	9.221	0.002	0.021	0.002~0.253

* control data.

**Table 3:** Receiver operating characteristic curve of augmented vertebral refracture after PKP in OVCFs.

	AUC	*p* Value	95% CI	Optimal Cutoff Value	Sensitivity	Specificity	Youden’s Index
Restoration of Cobb angle	0.863	<0.001	0.768~0.958	36.432	0.818	0.909	0.727
Absence of postoperative anti-osteoporosis treatment	0.760	<0.001	0.631~0.890	-	0.591	0.930	0.521
Logistic regression	0.977	<0.001	0.939~1.000		0.955	0.951	0.906

## Data Availability

The datasets used and analyzed during the current study are available from the corresponding author on reasonable request. The data are not publicly available because of privacy or ethical restrictions.
